# 环孢素A联合达那唑±沙利度胺治疗原始细胞不增高骨髓增生异常综合征的疗效及其影响因素分析

**DOI:** 10.3760/cma.j.issn.0253-2727.2021.05.005

**Published:** 2021-05

**Authors:** 喻堤 张, 泽锋 徐, 铁军 秦, 冰 李, 士强 曲, 丽娟 潘, 蒙 焦, 志坚 肖

**Affiliations:** 中国医学科学院北京协和医学院血液病医院（中国医学科学院血液学研究所），国家血液系统疾病临床医学研究中心，实验血液学国家重点实验室，天津 300020 State Key Laboratory of Experimental Hematology, National Clinical Research Center for Blood Diseases, Institute of Hematology & Blood Diseases Hospital, Chinese Academy of Medical Sciences & Peking Union Medical College, Tianjin 300020, China

**Keywords:** 骨髓增生异常综合征, U2AF1基因, 环孢素A, 达那唑, 疗效, Myelodysplastic syndrome, U2AF1 gene, Cyclosporine A, Danazol, Treatment outcome

## Abstract

**目的:**

探讨环孢素A（CsA）联合达那唑±沙利度胺治疗原始细胞不增高骨髓增生异常综合征（MDS）疗效及其影响因素。

**方法:**

收集2011年12月至2019年12月在中国医学科学院血液病医院确诊且接受CsA联合达那唑±沙利度胺治疗的115例原始细胞不增高初诊原发性MDS患者临床资料，回顾性分析其临床特征、疗效及疗效影响因素，并建立疗效预测模型。

**结果:**

55例（47.8％）患者获得治疗反应，其中11例获得完全缓解，44例获得血液学改善［红系反应率为49.5％（52/105），血小板反应率为40.7％（35/86），中性粒细胞反应率为35.0％（14/40）］。29例红细胞输注依赖的患者中7例（24.1％）脱离输血依赖。总体中位疗效持续时间为20（3～84）个月。单因素分析显示年龄<60岁较≥60岁患者疗效好（52.5％对22.2％，*P*＝0.018），而伴红细胞输注依赖较非红细胞输注依赖患者（24.1％对55.8％，*P*＝0.003）、U2AF1突变型患者较U2AF1野生型患者（26.1％对53.2％，*P*＝0.020）疗效差。多因素分析显示年龄<60岁（*OR*＝4.302, 95％*CI* 1.245～14.820，*P*＝0.021）、无红细胞输注依赖（*OR*＝3.774，95％*CI* 1.400～10.177，*P*＝0.009）和无U2AF1突变（*OR*＝3.414，95％*CI* 1.168～9.978，*P*＝0.025）均为获得血液学改善的独立预后因素。联合上述影响疗效的独立预后因素建立疗效预测模型，具有0、1、2、3个危险因素患者的总有效率分别为65％、30％～35％、10％～15％、3％。

**结论:**

CsA联合达那唑±沙利度胺可有效改善原始细胞不增高MDS患者的血细胞减少症状。年龄<60岁、不伴红细胞输注依赖且无U2AF1突变患者治疗反应较好。

## Abstract (en)

**Objective:**

To explore the outcome of cyclosporine A（CsA）combined with danazol with or without thalidomide regimen for myelodysplastic syndrome（MDS）with low-percentage bone marrow blasts and predictive factors for treatment response.

**Methods:**

Data of 115 subjects who were newly diagnosed with primary MDS with low-percentage bone marrow blasts and were treated with CsA combined with danazol with or without thalidomide from December 2011 to December 2019 in our center were collected. Their clinical features, efficacy, and predictive factors of efficacy were retrospectively analyzed. A model for predicting this response was developed.

**Results:**

A total of 55 subjects responded （47.8％）, including 11 complete responses and 44 hematologic improvements. Fifty-two patients（52/105, 49.5％）achieved erythrocyte response; 35（35/86, 40.7％）, platelet response; and 14（14/40, 35％）, neutrophil response. Of 29 subjects（24.1％）, 7 who were red blood cell（RBC）transfusion-dependent became independent of transfusion. The median response duration was 20 months（range, 3–84 months）. In the univariate analysis, patients <0 years had a higher response rate than those ≥60 years（52.5％ *vs* 22.2％, *P*＝0.018）. Contrarily, the response rate was substantially decreased in patients with RBC transfusion dependence compared with those without RBC transfusion dependence（24.1％ *vs* 55.8％, *P*＝0.003）, as well as in patients with the mutated U2AF1 compared with those with the wild-type U2AF1 （26.1％ *vs* 53.2％, *P*＝0.020）. In multivariable analyses, age <0 years（*OR*＝4.302, 95％ *CI* 1.245–14.820, *P*＝0.021）, RBC transfusion dependence（*OR*＝3.774, 95％ *CI* 1.400–10.177, *P*＝0.009）, and U2AF1 mutation （*OR*＝3.414, 95％ *CI* 1.168–9.978, *P*＝0.025） were significantly correlated with response. Variables that independently predicted the response were combined to generate the predictive model. According to the model, the overall response rates of patients with 0, 1, 2, and 3 risk factors were 65％, 30％–35％, 10％–15％, and 3％, respectively.

**Conclusion:**

CsA combined with danazol with or without thalidomide regimen could improve cytopenia symptoms in patients with MDS with lowpercentage bone marrow blasts. At age <60 years, no transfusion dependence of RBC and wild-type U2AF1 mutation is a favorable prognostic factor.

骨髓增生异常综合征（MDS）是一组异质性髓系肿瘤，与再生障碍性贫血、阵发性睡眠性血红蛋白尿症等同归于骨髓衰竭综合征[1]。免疫抑制治疗（IST）可有效改善部分MDS患者的血细胞减少[2]，但影响疗效的因素及哪些患者适合选择IST尚无共识。此前，我们报告了环孢素A（CsA）联合沙利度胺治疗MDS的疗效及远期疗效影响因素[3]–[4]，本研究对CsA联合达那唑±沙利度胺治疗原始细胞不增高MDS的疗效及其疗效影响因素进行回顾性分析。

## 病例与方法

1. 病例资料：本研究纳入标准：①符合WHO（2016）MDS诊断标准[5]；②骨髓原始细胞比例<5％，且外周血原始细胞比例<2％；③年龄≥18岁；④规律接受CsA联合达那唑±沙利度胺方案治疗至少12周，可进行疗效及不良反应评价；⑤前期未接受除血制品输注之外的治疗；⑥东部肿瘤协作组（ECOG）体能状态评估标准0～2级；⑦无肝、肾等重要脏器功能严重异常。2011年12月至2019年12月于我院MDS诊疗中心就诊的115例患者纳入研究。男67例（58.3％），中位年龄48（21～77）岁。初诊时中位HGB为74（31～146）g/L，中位ANC为1.23（0.06～7.39）×10^9^/L，中位PLT为39（5～402）×10^9^/L，中位骨髓原始细胞比例为0.5％（0～4.5％）。WHO（2016）MDS分型：MDS伴单系血细胞发育异常（MDS-SLD）3例（2.6％），MDS伴环状铁粒幼红细胞（MDS-RS）5例（4.4％），MDS伴多系血细胞发育异常（MDS-MLD）104例（90.4％），MDS不能分类（MDS-U）3例（2.6％）。采用修订的国际预后积分系统（IPSS-R）[6]对患者进行预后分组：极低危4例（3.5％），低危43例（34.7％），中危55例（47.8％），高危11例（9.6％），极高危2例（1.7％）。29例（25.2％）患者治疗前红细胞输注依赖。65例（56.5％）患者接受CsA联合达那唑和沙利度胺治疗，50例（43.5％）患者接受CsA联合达那唑治疗。两组患者各项基线特征差异均无统计学意义（*P*值均>0.05）。

2. 治疗方法：CsA的起始剂量为3 mg·kg^−1^·d^−1^，用药2周后调整剂量使CsA血清谷浓度维持在100～200 µg/L，最终CsA剂量为3～5 mg·kg^−1^·d^−1^。达那唑600 mg/d（分3次口服）。沙利度胺50 mg/d，每日睡前口服。治疗24周后未获得血液学改善（HI）则改用其他治疗方案。治疗有效的患者如无3级以上药物相关不良反应，持续用药至HI后疾病进展或复发。

3. 靶向测序检测基因突变：取患者诊断时的骨髓标本分离单个核细胞，提取DNA。采用Ion Torrent半导体测序平台进行测序。具体方法参见本研究组此前已发表文献[7]。

4. 疗效判定及不良反应评价标准：参照国际工作组（IWG）标准[8]判定疗效。治疗12周后进行疗效评价，若未达到HI则延续到24周进行评价。若24周后患者仍未达HI，判定为无效。红细胞输注依赖定义为每月输注红细胞>2 U，持续超过3个月。脱离红细胞输注依赖的定义为至少3个月不需要输注红细胞[9]。不良反应分级依据美国国立肿瘤研究所常见毒性标准（version 4.0）进行判定。

5. 随访：所有患者均随访至2020年6月30日，中位随访时间为21（4～98）个月。随访资料来源于住院病历、门诊病历及电话随访记录。对随访期间死亡的病例，按照病例记录或与患者家属电话联系确认。总生存（OS）期按诊断日期到死亡或末次随访日期计算。

6. 统计学处理：应用SPSS 23.0软件进行统计分析。非正态分布的计量资料采用Mann-Whitney *U*检验，数据以“中位数（范围）”表示。分类资料采用卡方检验或Fisher精确概率法进行差异性分析。采用Kaplan-Meier法绘制生存曲线并通过Log-rank检验进行单因素分析，采用Cox比例风险回归模型进行多因素分析。采用Logistic回归对影响疗效的临床和实验室特征进行多因素分析并构建预测模型。双侧检验*P*<0.05为差异有统计学意义。采用ROC曲线对疗效预测模型性能进行评估。

## 结果

一、疗效评价

全部115例患者中共55例（47.8％）治疗有效，包括11例完全缓解（CR）和44例HI。CsA联合达那唑和沙利度胺组患者与CsA联合达那唑组患者总有效率（ORR）（43.1％对55.0％，*P*＝0.245）、红系反应（HI-E）率（44.1％对56.5％，*P*＝0.200）、血小板反应（HI-P）率（34.8％对47.5％，*P*＝0.122）及中性粒细胞反应（HI-N）率（25.0％对40.0％，*P*＝0.074）差异均无统计学意义。105例患者治疗前HGB<110 g/L，其中52例（49.5％）获得HI-E。29例患者治疗前红细胞输注依赖，其中7例（24.1％）获得HI-E且脱离红细胞输注依赖。86例患者治疗前PLT<100×10^9^/L，其中35例获得HI-P（40.7％）。40例患者治疗前ANC<1×10^9^/L，其中14例获得HI-N（35.0％）。中位起效时间为12（3～24）周。获得最佳疗效的中位时间为24（12～40）周。总体中位疗效持续时间为20（3～84）个月；HI-E中位疗效持续时间为19（3～84）个月；HI-P中位疗效持续时间为27（3～84）个月；HI-N中位疗效持续时间为23（4～66）个月。36例患者在治疗过程中出现疾病进展，其中11例在获得HI后出现疾病复发或进展。

二、疗效影响因素分析

1. 临床和实验室特征对疗效的影响：对年龄、性别、血细胞计数、骨髓原始细胞比例、骨髓增生程度、IPSS-R预后分组、WHO诊断分型、是否红细胞输注依赖、是否伴8号染色体三体（+8）及是否伴阵发性睡眠性血红蛋白尿（PNH）克隆等因素对疗效的影响进行单因素分析，结果示疗效与性别（*P*＝0.126）、HGB（*P*＝0.965）、PLT（*P*＝0.134）、ANC（*P*＝0.491）、骨髓原始细胞比例（*P*＝0.314）、骨髓增生程度（*P*＝0.079）、IPSS-R预后分组（*P*＝0.404）、WHO诊断分型（*P*＝0.625）、是否伴+8（*P*＝0.205）、是否伴PNH克隆（*P*＝0.093）、细胞遗传学分组（*P*＝0.202）无明显相关性；而年龄和是否伴红细胞输注依赖对疗效影响显著：年龄<60岁组患者ORR高于年龄≥60岁组患者（52.5％对22.2％，*P*＝0.018）；不伴红细胞输注依赖组患者ORR高于红细胞输注依赖组患者（55.8％对24.1％，*P*＝0.003）（表1）。

**表1:** 影响环孢素A（CsA）联合达那唑±沙利度胺治疗骨髓增生异常综合征（MDS）疗效的单因素分析

影响因素	治疗有效组（55例）	治疗无效组（60例）	*P*值
年龄≥60岁［例（％）］	4（7.3）	14（23.3）	0.018
男性［例（％）］	28（50.1）	39（65.0）	0.126
HGB［g/L，*M*（范围）］	75.5（31~125）	73（40~146）	0.965
ANC［×10^9^/L，*M*（范围）］	1.18（0.06~9.13）	1.29（0.19~7.39）	0.491
PLT［×10^9^/L，*M*（范围）］	29（5~367）	48（7~402）	0.134
骨髓原始细胞比例［％，*M*（范围）］	0.5（0~3.5）	0.75（0~4.5）	0.314
骨髓增生低下［例（％）］	31（56.4）	24（40.0）	0.079
IPSS-R预后分组［例（％）］			0.404
极低危	2（3.6）	3（5.0）	
低危	20（36.4）	24（40.0）	
中危	22（40.0）	25（41.7）	
高危	11（20.0）	6（10.0）	
极高危	0（0.0）	2（3.3）	
WHO诊断分型［例（％）］			0.625
MDS-SLD	1（1.8）	2（3.3）	
MDS-RS	2（3.6）	3（5.0）	
MDS-MLD	50（90.9）	54（90.0）	
MLD-U2	（3.6）	1（1.7）	
红细胞输注依赖［例（％）	］7（12.7）	22（36.7）	0.003
是否伴+8［例（％）］	10（18.2）	6（10.0）	0.205
细胞遗传学分组［例（％）］			0.202
极好	1（1.8）	1（1.7）	
好	36（65.5）	41（68.3）	
中等	14（25.5）	13（21.7）	
差	4（7.3）	1（1.7）	
极差	0（0.0）	4（6.7）	
PNH克隆阳性［例（％）］	6（10.9）	1（1.7）	0.093
基因突变［例（％）］			
U2AF1	6（10.9）	17（28.3）	0.020
ASXL1	3（5.5）	9（15.0）	0.094
DNMT3A	3（5.1）	5（9.1）	0.621
SF3B1	3（5.5）	4（6.7）	1.000
TET2	3（5.5）	3（5.0）	1.000
SETBP1	2（3.7）	4（6.7）	0.774
TP53	2（3.6）	3（5.0）	1.000
RUNX1	1（1.8）	3（5.0）	0.674
治疗方案［例（％）］			0.245
CsA+达那唑+沙利度胺	28（50.9）	32（53.3）	
CsA+达那唑	27（49.1）	28（46.7）	

注：IPSS-R：修订版国际预后积分系统；MDS-SLD：MDS伴单系血细胞发育异常；MDS-RS：MDS伴环状铁粒幼红细胞；MDS-MLD：MDS伴多系血细胞发育异常；MDS-U：MDS不能分类；PNH：阵发性睡眠性血红蛋白尿症

2. 基因突变对疗效的影响：115例患者平均携带基因突变数目为0.678个，53例（46.1％）携带≥1个基因突变。U2AF1为最常见的基因突变（23例，20％），其中S34F 13例（65.2％），S34Y 8例（34.8％），中位等位基因变异频率（VAF）为31.19％（5.10％～51.22％）。其余基因突变检出率依次为ASXL1（12例，10.4％），DNMT3A（8例，7.0％），SF3B1（7例，6.1％），TET2、SETBP1（6例，5.2％），TP53（5例，4.3％），RUNX1（4例，3.5％），SRSF2（2例，1.7％），NPM1、CSF3R、NOTCH1、CEBPA和FLT3（1例，0.9％）（图1）。对突变频率>2％基因对疗效的影响进行单因素分析，结果显示U2AF1突变对疗效影响显著：U2AF1野生型组患者ORR显著高于U2AF1突变型组患者（53.2％对26.1％，*P*＝0.020）；除U2AF1外其他基因突变与疗效均无明显相关性（*P*>0.05）（表1）。

**图1:**
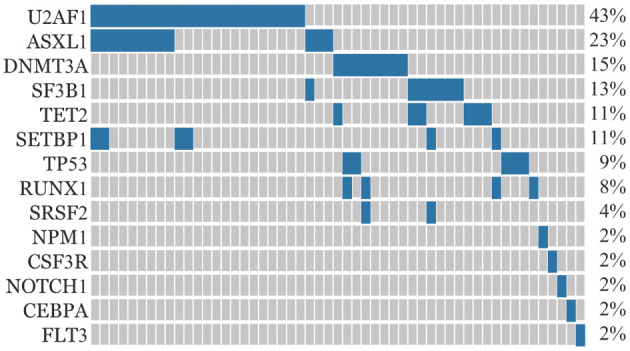
115 例骨髓增生异常综合征患者基因突变谱系图

3. 影响疗效的多因素分析：将单因素分析中*P*<0.1的因素纳入多因素Logistic回归模型，结果示年龄<60岁（*OR*＝4.302，95％*CI* 1.248～14.827，*P*＝0.021），无红细胞输注依赖（*OR*＝3.774，95％*CI* 1.400～10.177，*P*＝0.009）和无U2AF1突变（*OR*＝3.414，95％*CI* 1.168～9.978，*P*＝0.025）均为获得HI的独立预后因素。联合上述影响疗效的独立预后因素，建立疗效预测的可视化图形（图2）：年龄<60岁、无红细胞输注依赖且无U2AF1突变的患者疗效最好（ORR＝65.75％），具备其中1个危险因素ORR为30％～35％，具备其中2个危险因素ORR为10％～15％，具备3个危险因素（年龄≥60岁、红细胞输注依赖且U2AF1突变）患者疗效最差，ORR仅为3.35％。采用ROC曲线对该疗效预测模型进行评估：AUC＝0.711（95％ *CI* 0.624～0.797）。

**图2:**
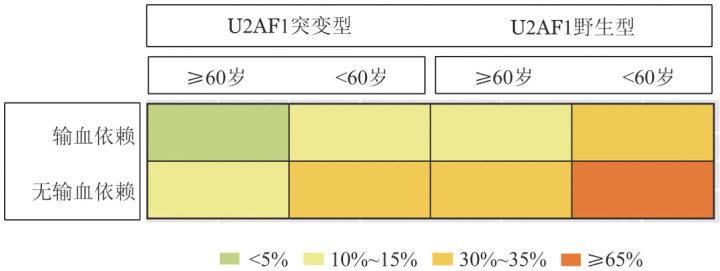
环孢素A联合达那唑±沙利度胺治疗原始细胞不增高的骨髓增生异常综合征的疗效预测模型

四、不良反应

治疗过程中12例（10.4％）患者出现1、2级转氨酶升高，经保护肝细胞治疗或调整药物剂量后好转。6例（5.2％）患者出现1、2级血清肌酐增高，调整CsA剂量或停药后肾功能恢复正常。其他治疗相关不良反应包括便秘（8例，7.0％）、齿龈增生（4例，3.5％）、皮疹（4例，3.5％）、水肿（3例，2.6％）、肢端麻木（2例，1.7％），均为1级，经对症处理后可耐受。未发生3级以上不良反应。

五、生存分析及影响生存的因素

截至末次随访，共26例（22.6％）患者死亡，其中6例（5.2％）转化为急性髓系白血病（转白）后死亡，20例（17.4％）死于骨髓衰竭（出血或感染）。所有患者中位OS期未达到，3年OS率为70.4％（95％ *CI* 65.1％～75.9％）。单因素分析结果示IPSS-R危险分组（*P*<0.001）和疗效（*P*<0.001）是影响MDS患者OS的因素（表2）。将单因素分析*P*<0.1的因素纳入COX回归分析模型后，未获得治疗反应（*HR*＝7.502，95％ *CI* 2.281～24.669，*P*＝0.001）和IPSS-R较高危组（*HR*＝2.110，95％*CI* 1.350～3.298，*P*＝0.001）是影响患者OS的独立危险因素（表2）。

**表2:** 影响骨髓增生异常综合征患者总生存的单因素及多因素分析

影响因素	单因素分析		多因素分析	
	*HR*（95％ *CI*）	*P*值	*HR*（95％ *CI*）	*P*值
年龄>60岁	2.224（0.939～5.361）	0.069	1.278（0.471～3.465）	0.630
IPSS-R较高危组	3.014（1.207～7.522）	0.018	2.110（1.350～3.298）	0.001
红细胞输注依赖	2.257（1.001～5.081）	0.050	1.065（0.398～2.847）	0.871
PNH克隆阳性	0.427（0.445～3.288）	0.412	–	–
骨髓增生正常或活跃	0.437（0.190～1.005）	0.051	1.532（0.577～4.070）	0.378
U2AF1突变	0.469（0.203～1.085）	0.077	1.076（0.386～3.003）	0.888
治疗无效	7.343（2.505～21.526）	0.001	7.502（2.281～24.669）	0.001

注：IPSS-R：修订版国际预后积分系统；PNH：阵发性睡眠性血红蛋白尿症；^a^IPSS-R总分≤3.5分为较低危组，IPSS-R总分>3.5分为较高危组；–：不适用

## 讨论

MDS患者的自然病程和预后差异较大，宜个体化选择治疗方案。对于骨髓原始细胞比例<5％且出现血细胞减少症状的非5q–综合征患者，如不适合采用细胞因子治疗或对细胞因子治疗反应不佳，免疫调节剂和IST为推荐的治疗方案[10]。

Epling-Burnette等[11]研究表明MDS患者体内过度激活的T细胞选择性地免疫攻击造血系统是MDS患者骨髓功能衰竭的原因之一。抑制寡克隆或单克隆扩增的T细胞是IST治疗MDS的理论基础。抗胸腺细胞球蛋白（ATG）±CsA的IST对较低危MDS的有效率为20％～50％[12]–[13]。沙利度胺具多效免疫调节机制，可改善MDS患者血细胞减少症状，且对原始细胞不增高的MDS患者疗效更好[14]。其单药治疗MDS有效率为9％～56％[14]–[15]。我中心既往CsA联合沙利度胺治疗MDS患者HI率为53％[3]–[4]。

达那唑是人工合成的类雄激素药物，其雄性化副作用弱，是国际上应用最广泛的人工合成雄激素，单药治疗MDS有效率为40％～60％，尤其能显著提高MDS患者的血小板水平[16]–[17]。早期研究证明达那唑具免疫调节作用，可纠正辅助性CD4^+^ T细胞与效应性CD8^+^ T细胞的比例失衡，并抑制TNF-α和IL-1β的生成，从而促进造血[18]–[19]。近期研究发现其改善造血的机制可能还与延缓端粒缩短有关[20]。

本研究初步评估了CsA联合达那唑±沙利度胺治疗原始细胞不增高MDS的有效性，结果示所有患者ORR为47.8％，而CsA联合达那唑和沙利度胺组患者与CsA联合达那唑组患者ORR（43.1％对55.0％，*P*＝0.245）及各系有效率差异均无统计学意义。达那唑可通过其免疫调节功能协同CsA纠正MDS患者体内免疫紊乱状态，在此基础上，考虑沙利度胺不能进一步协同CsA联合达那唑发挥作用，从而提高疗效，但尚不能除外由于病例数尚少、研究对象的选择偏倚等原因造成结果偏差。

如何识别可能从IST受益的患者一直是临床难题。既往研究[13],[21]–[23]定义可以预测疗效的因素包括：年轻（<60岁）、骨髓增生低下、HLA-DR15位点阳性、依赖血制品输注时间较短、PNH克隆阳性、较高的CD8/CD4 T细胞比值等。Saunthararaja等[21]基于年龄、依赖血制品输注时间和HLA-DR15基因型建立了评估MDS患者对IST反应率的预测模型。但该模型在此后Lim等[23]的研究中未能得到验证。本研究证实了年龄（*P*<0.021）和红细胞输注依赖（*P*＝0.009）对疗效的预测价值。其他因素在本研究中未能得到验证，考虑原因可能为：①各研究纳入研究对象的标准不同；②检测方法及判断标准（如PNH克隆的检测方法、PNH克隆阳性的判断标准等）存在差异；③均为回顾性分析，且入组病例数各异。此外，本研究中2例IPSS-R极高危组患者均未获得治疗反应且在治疗过程中出现疾病进展。IPSS-R分组并非反应免疫状态的指标，本研究中疗效与IPSS-R危险分组亦无明显相关性，但IPSS-R极高危组患者相对生存时间短、转白风险高。因此我们考虑可能这部分患者从IST获益有限而需要更积极的治疗。

迄今，很少有研究报道MDS中基因突变对IST疗效的影响。近期Zhang等[24]报道了SF3B1突变是影响MDS患者IST疗效的独立危险因素，但我们的研究结果不支持这一结论。本队列中出现的所有突变基因均纳入单因素分析，仅U2AF1突变与疗效显著相关（*P*＝0.025），其他基因突变与疗效均无明显相关性（*P*>0.05）。造成研究结果差异的原因可能是各项研究病例数均相对较少以及入组病例各亚型病例数不同。此外，不同种族MDS患者的基因突变谱系不尽相同[25]，我们既往的研究显示，U2AF1突变是中国MDS患者中最常见的基因突变[26]，而西方人群中MDS患者SF3B1基因的突变频率高于U2AF1[27]。U2AF1突变导致疗效不佳的原因目前尚不清楚。Smith等[28]研究显示U2AF1突变会激活白细胞介素-1受体相关激酶4（IRK4）异构体，从而导致NF-κB和促分裂原活化蛋白激酶（MAPK）信号通路的过度激活，因此，我们推测U2AF1突变对IST疗效的影响可能与IRAK4的激活有关。

IST长程抑制T细胞功能，可致MDS克隆免疫逃逸而异常扩张。因此理论上IST可能增加MDS患者的转白风险。然而Sloand等[29]对应用IST的MDS患者进行长期随访，结果表明IST有效的患者不仅可以获得长期的HI，且较年龄和国际预后积分系统（IPSS）危险评分匹配的未治MDS患者，OS期更长，转白风险更低。IST无效的患者转白风险亦不高于匹配的MDS患者。既往Greenberg等[30]报道的未治MDS患者总体转白率为22.1％，本研究中MDS患者转白率为5.2％，但本研究仅纳入原始细胞不增高的MDS患者且随访时间相对较短。因此从长远来看，IST对MDS患者而言仍是安全的治疗选择。

综上，本研究肯定了CsA联合达那唑±沙利度胺治疗原始细胞不增高MDS患者的有效性和安全性，证实了年龄和红细胞输注依赖对疗效的预测价值，同时首次提出U2AF1基因突变是影响疗效的独立危险因素。基于上述结果我们提出一项疗效预测模型，经模型选择的MDS患者ORR最高可达65％，可为MDS患者治疗方案的选择提供参考。但本研究为单中心回顾性研究，样本量相对较少，且尚缺乏对U2AF1突变影响治疗效果的基础研究，仍需多中心前瞻性临床研究和基础研究进一步验证我们的结论。
